# Evaluation of red blood cell transfusion threshold in the management of brain-dead organ donors

**DOI:** 10.1097/MD.0000000000032353

**Published:** 2022-12-16

**Authors:** Sungjeep Kim, Kyunghak Choi, Min Ae Keum, Min Soo Kim, Sun Geon Yoon, Kyu-Hyouck Kyoung

**Affiliations:** a Department of Trauma Surgery, Ulsan University Hospital, University of Ulsan College of Medicine, Ulsan, Korea; b Department of Neurological Surgery, Ulsan University Hospital, University of Ulsan College of Medicine, Ulsan, Korea.

**Keywords:** brain-dead organ donors, graft function, hemoglobin concentration, organ transplantation, transfusion threshold

## Abstract

The disparity between the demand and supply of organs has necessitated an expansion of the criteria for organ donation. Consequently, numerous guidelines have been proposed for managing brain-dead organ donors (BDODs) to improve their organ function and the organ procurement rate. Therefore, we aimed to evaluate the previously recommended threshold for red blood cell transfusion in BDODs. Medical records of BDODs were retrospectively reviewed from January 2012 to December 2021. We enrolled BDODs who stayed for more than 24 hours at an hospital organ procurement organization. We analyzed their organ function and the rate of organ procurement according to the hemoglobin concentration. A total of 111 BDODs were enrolled and divided into the following 2 groups: hemoglobin (Hb) ≥ 10 g/dL (45.0 %) and Hb < 10 g/dL (55.0 %). There were no significant differences between the groups in the total bilirubin, creatinine, arterial blood lactate, and the rate of organ procurement. A correlation analysis did not reveal any association between the hemoglobin concentration and organ function of the BDODs. Hemoglobin concentration of 10 g/dL cannot be considered a threshold for red blood cell transfusion. Furthermore, organ function is not correlated with a hemoglobin concentration > 7 g/dL. Restrictive transfusion strategy is appropriate for BDOD management.

## 1. Introduction

The discrepancy between demand and supply for transplantable organs has been steadily increasing. Researchers have expanded the criteria for organ donation to compensate for organ shortage, additionally proposing numerous guidelines for managing brain-dead organ donors (BDODs) to improve their organ function and the organ procurement rate.

Strategies for shock resuscitation are centered around the need to enhance tissue perfusion and oxygen delivery regardless of its causes. Hemoglobin plays a key role in oxygen delivery^[1]^;thus, the transfusion threshold has been identified as a parameter to maintain optimal hemoglobin concentration. The Transfusion Requirements in Critical Care (TRICC) trial reported that restrictive transfusion (threshold at hemoglobin < 7.0 g/dL) was superior to liberal transfusion (threshold at hemoglobin < 10.0 g/dL) in 1999.^[2]^ Thus, subsequent studies have established a hemoglobin concentration of 7.0 g/dL as the threshold for red blood cell (RBC) transfusion.^[3,4]^

Guidelines for the management of BDODs consist of ventilator care, hemodynamic support, hormonal resuscitation, and the correction of metabolic derangement; however, most goals were derived from studies on patients experiencing shock and not BDODs. A recommended level of hemoglobin concentration has been inferred from shock resuscitation studies without verification on the BDOD population, despite a substantial effect on organ function. In this study, we aimed to investigate the effectiveness of hemoglobin concentration on organ function in BDODs.

## 2. Materials and Methods

### 2.1. Participants

The medical records of BDODs were reviewed and analyzed from January 2012 to December 2021. This study was approved by the institutional review board, which waived the need for informed consent as the patients did not undergo additional interventions.

We enrolled BDODs who stayed at the intensive care unit of the hospital organ procurement organization (HOPO) for more than 24 hours. Individuals under 16 years of age and with liver cirrhosis or end-stage renal disease were excluded to avoid bias in the laboratory data and the rate of organ procurement.

### 2.2. Measurements

The hemoglobin concentration was determined with the mean value within 24 hours before organ procurement. We evaluated liver and kidney function based on the concluding value of the total bilirubin and serum creatinine. We measured the influence of hemoglobin concentration on the severity of BDODs’ condition through the concluding values of arterial blood lactate and the vasoactive-inotropic score (VIS). VIS was calculated as follows: dopamine dose (µg/kg/minute) + dobutamine dose (µg/kg/minute) + 100 × epinephrine dose (µg/kg/minute) + 10 × milrinone dose (µg/kg/minute) + 10,000 × vasopressin dose (U/kg/minute) + 100 × norepinephrine dose (µg/kg/minute).

BDODs were divided into 2 groups namely, hemoglobin (Hb) ≥ 10 and Hb < 10, according to hemoglobin concentrations ≥ 10.0 and < 10.0 g/dL to evaluate the reliability of the 10 g/dL-threshold on the outcomes. We performed a correlation analysis to evaluate the relationship between the hemoglobin concentration and outcomes above concentration ≥ 7 g/dL.

### 2.3. Statistical analysis

Continuous variables are presented as means ± standard deviations, and all data were analyzed using SPSS 24 (IBM, New York, USA). Categorical and continuous variables were analyzed using a chi-square test or Fischer’s exact test and Student’s *t* test, respectively. We evaluated the correlation among the variables using Pearson’s correlation analysis and Spearman’s correlation analysis. Statistical significance was defined as a 2-sided *P* value < .05.

## 3. Results

**Figure 1.**
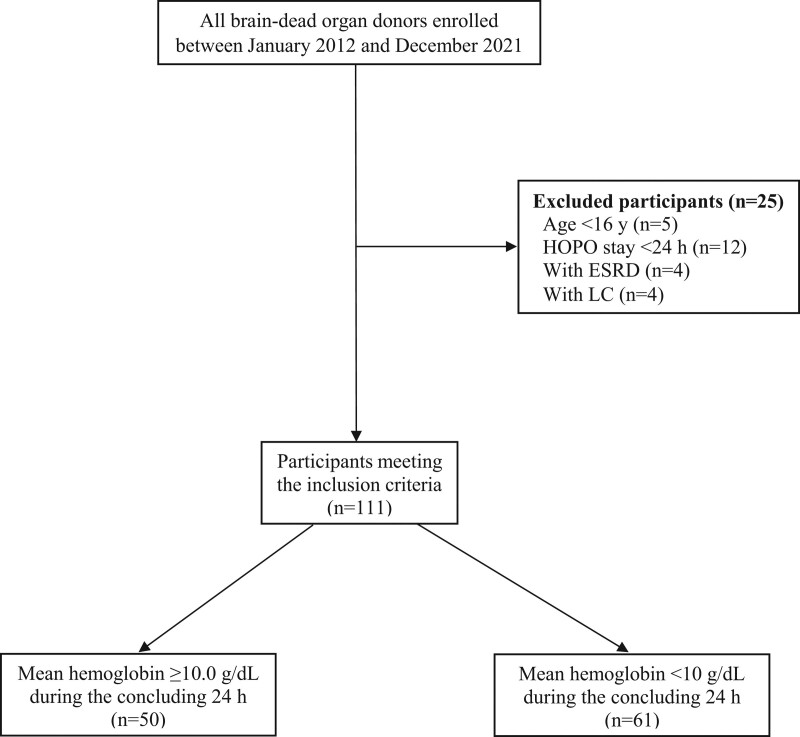
Flowchart for patient enrollment. ESRD = end-stage renal disease, HOPO = hospital organ procurement organization, LC = liver cirrhosis.

A total of 136 BDODs underwent organ procurement, of which we enrolled 111 BDODs. Twenty-five BDODs were excluded owing to their age, <24-hours stay at the HOPO, end-stage renal disease, and liver cirrhosis (Fig. 1).

Fifty (45.0%) BDODs were classified into the Hb ≥ 10 group, whereas 61 (55.0%) were classified into the Hb < 10 group. There were no significant differences in the baseline characteristics, such as age, sex, causes of brain death, and surgery (Table 1).

**Table 1:** Baseline characteristics of BDODs.

Characteristic	Mean Hemoglobin ≥ 10.0 g/dL	Mean Hemoglobin < 10 g/dL	*P* value
**Number (%**)	50 (45.0)	61 (55.0)	
**Age, yr**	51.2 ± 12.9	48.8 ± 13.6	.343
**Male sex, n (%**)	38 (76.0)	38 (62.3)	.122
**Body mass index (kg/m**^**2**^)	23.5 ± 3.4	23.8 ± 3.7	.676
**Mean hemoglobin (g/dL**)	11.3 ± 1.3	9.0 ± 0.6	<.001
**Medical history, n (%**)			.801
Diabetes	6 (12.0)	6 (9.8)	
Hypertension	13 (26.0)	15 (24.6)	
Cardiocerebrovascular disease	5 (10.0)	2 (3.3)	
Other	5 (10.0)	6 (9.8)	
**Causes of brain death, n (%**)			.094
Traumatic brain injury	14 (28.0)	25 (41.0)	
Spontaneous brain hemorrhage	30 (60.0)	24 (39.3)	
Hypoxic brain injury	6 (12.0)	12 (19.7)	
**Referred for organ procurement, n (%**)	17 (34.0)	11 (18.0)	.054
**Duration before organ procurement (day**)	9.9 ± 6.1	13.2 ± 11.3	.065
**Neurosurgery or intervention, n (%**)	27 (54.0)	39 (63.9)	.289
**Non-neurosurgery, n (%**)	2 (4.0)	3 (4.9)	1.000

BDODs = brain-dead organ donors.

Regarding outcome measurement, the VIS was significantly higher in the Hb ≥ 10 group (15.3 ± 14.5 vs 6.6 ± 8.1, *P < *.001); however, there were no differences in the arterial blood lactate, total bilirubin, and creatinine. The rate of organ procurement in each organ, as well as the number of procured organs, did not differ between the groups (Table 2).

**Table 2:** Outcomes of BDODs.

Characteristic	Mean Hemoglobin ≥ 10.0 g/dL	Mean Hemoglobin < 10 g/dL	*P* value
**Vasoactive inotropic score, median (IQR**)	11.0 (5.0–21.3)	4.0 (0–9.3)	<.001
**Arterial blood lactate (mmol/L**)	1.12 ± 0.61	1.26 ± 1.52	.526
**Total bilirubin (mg/dL**)	1.34 ± 1.36	1.55 ± 1.74	.474
**Creatinine (mg/dL**)	1.15 ± 0.88	1.57 ± 1.71	.100
**The rate of organ procurement, n (%**)			
Heart	11 (22.0)	16 (26.2)	.605
Lung	1 (2.0)	4 (6.6)	.376
Liver	45 (90.0)	55 (90.2)	1.000
Pancreas	3 (6.0)	5 (8.2)	.728
Kidney	49 (98.0)	59 (96.7)	1.000
The number of procured organ	2.2 ± 0.7	2.3 ± 0.8	.519

A pair of kidneys from 1 brain-dead organ donor were considered as 1 organ.

BDODs = brain-dead organ donors, IQR = interquatile range.

**Figure 2.**
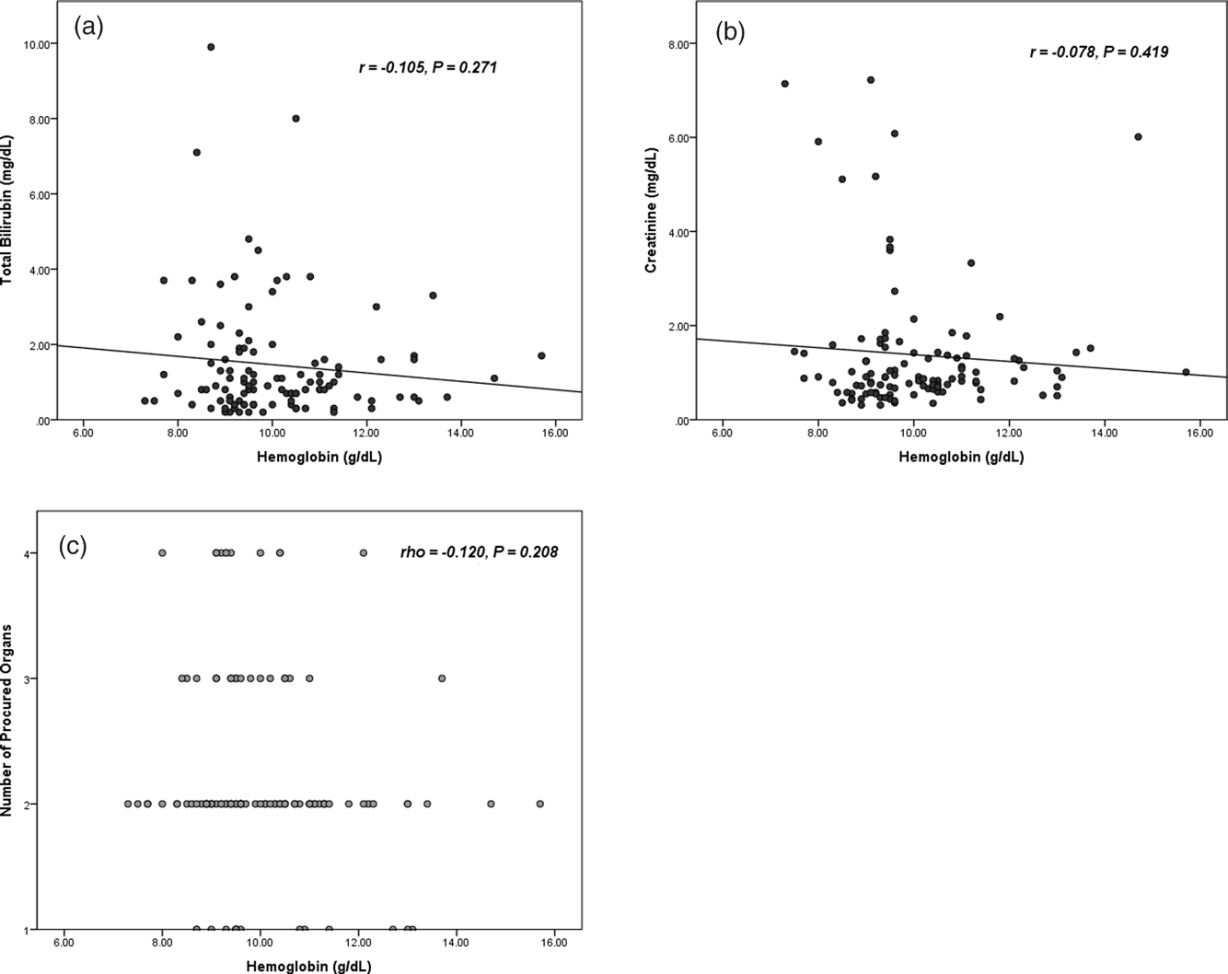
The correlation between hemoglobin concentration and outcomes. A and B have been analyzed using Pearson’s correlation analysis. C has been analyzed using Spearman’s correlation analysis.

All BDODs displayed a mean hemoglobin concentration ≥ 7 g/dL. The hemoglobin concentration was not correlated with the total bilirubin and creatinine. The number of procured organs was not correlated with the hemoglobin concentration (Fig. 2).

## 4. Discussion

When organ donation is decided upon, the goal of treatment is to improve the quality of the graft from the donor. Despite reports of favorable outcomes following transplantation using low quality grafts, organ dysfunction of BDODs has been a major cause of delayed graft function or graft discard.^[5–7]^

The correction of anemia plays an important role in organ function via improvement in the oxygen-carrying capacity; therefore, this principle has been extended to BDOD management. For several decades, clinicians have considered the traditional 10/30 rule (hemoglobin 10 g/dL, hematocrit 30%) as an optimal transfusion trigger;^[8,9]^ here, the guidelines for BDODs have recommended maintaining the hemoglobin concentration above 10 g/dL.^[10,11]^ The transfusion threshold in BDODs has been gradually shifted toward a hemoglobin concentration of 7 g/dL since the TRICC trial demonstrated the superiority of restrictive transfusion strategy.^[3,4,12]^ However, studies on transfusion threshold, including the TRICC trial, were conducted in critically ill patients and not BDODs, therefore, guidelines for the transfusion threshold for the BDOD population has not been established.

Although restrictive transfusion strategy has been widely accepted, RBC transfusion was required in 63.2% and 15.8% of BDODs with trough hemoglobin concentrations of > 8 g/dL and > 10 g/dL, respectively, in a study that demonstrated the actual practice of the transfusion.^[13]^ The various organs of the body have different susceptibility to anemia and different requirements for hemoglobin concentration in specific situations with varying risk for hypoperfusion, such as acute coronary syndrome, severe hypoxemia, acute hemorrhage, and hyperlactatemia.^[14–17]^ As a large number of BDODs who are critically ill are vulnerable to hypoperfusion, physicians tend to choose the liberal transfusion strategy, which leads to excessive transfusion.

In this study, we attempted to evaluate the reliability of the previously recommended threshold for RBC transfusion and demonstrated that there was no difference in organ function according to the hemoglobin concentration. Furthermore, no difference in the arterial blood lactate signified that higher hemoglobin levels did not improve oxygen delivery. Our result was consistent with previous studies, which indicate no positive effects of transfusion on the organ function and prognosis,^[18,19]^ and can provide supporting data to the guidelines suggesting the lack of evidence-based recommendations of the transfusion threshold in BDOD management.^[3,20]^ Considering that no hemorrhagic event occurred during the concluding 24 hours, a higher VIS in the Hb ≥ 10 group suggested that physicians maintained a higher hemoglobin concentration owing to the instability of vital signs, following traditional guidelines.

The application of an appropriate threshold for transfusion not only plays a vital role in reducing excessive transfusion but also in avoiding transfusion-related complications. There is no obvious evidence for the worsening outcomes of transplantation by immunologic adverse effects associated with RBC transfusion. Conversely, a large prospective observational study demonstrated that transfusion in BDODs exerted a protective effect on the initial renal graft function through unclear mechanisms.^[21]^

In terms of transplantation, adverse effects of transfusion are a problem of the donor-side with a directly aggravating condition rather than the recipient-side from the immunologic consequences of transfusion. Transfusion-related immunomodulation makes BDODs substantially prone to infections. It can be caused by marginal transfusion and displays a dose-dependent increase in infection risk.^[22–24]^ Despite rare donor-to-recipient transmission of infection and no differences in the recipient outcomes,^[25–27]^ infection can increase the burden of septic shock in BDODs. Aggravated shock condition because of sepsis requires greater vasopressor use and aggravates organ function, thus worsening the graft quality and the eventual rate of organ yield.

The outcome of lung transplantation is most susceptible to donor transfusion. Donor transfusion can injure the lung parenchyma through transfusion-related acute lung injury, besides resulting in detrimental gas exchange by transfusion-associated circulatory overload. Furthermore, these complications lead to primary graft dysfunction and recipient mortality.^[28–30]^ We did not investigate the influence of transfusion on recipient prognosis; nonetheless, our findings provided the evidence for justifying the restriction of transfusion at a hemoglobin concentration of 7 g/dL.

Furthermore, transfusion can cause laboratory errors. Serologic screening tests have been performed in all donors to prevent transmission of virulent pathogens, such as human immunodeficiency virus, hepatitis B virus, and hepatitis C virus, through transplantation. Large volumes of intravenous fluids and transfusion may result in false negative serological tests.^[31]^ Non-organ donor DNA acquired from transfusion can interrupt the reliability of human leukocyte antigen typing. It plays a vital role in organ allocation and outcome; therefore, the misinterpretation of human leukocyte antigen typing causes disastrous results.^[32]^

Our study had several limitations. First, the retrospective nature and relatively small cohort size of our single center study limited the statistical power. Second, we analyzed organ functions and concluding 24-hours hemoglobin; thus, we could not evaluate the effects of hemoglobin concentration change prior to the concluding 24 hours and could not obtain information on previous transfusions at other hospitals. Nonetheless, numerous BDODs are transferred to an HOPO for organ donation for a short period. Therefore, our findings can offer pragmatic suggestions for the management of BDODs. Third, we could not analyze the heart and lung function because laboratory assessment of these organs was not usually performed on the concluding day following the declaration of brain death. Fourth, all BDODs had a hemoglobin concentration ≥ 7 g/dL in the concluding 24 hours because organ procurement had been performed after the resuscitation period in most cases. Thus, we could not perform an analysis to investigate the eligibility of the hemoglobin concentration of 7 g/dL as the threshold of transfusion.

To our knowledge, this is the first study to evaluate the threshold of RBC transfusion in the BDOD population. Further prospective studies are required to identify the optimal transfusion threshold to optimize graft function.

## 5. Conclusion

A hemoglobin concentration of 10 g/dL cannot be considered as the threshold for RBC transfusion and organ function is not correlated with a hemoglobin concentration in the range of > 7 g/dL. Restrictive transfusion strategy can be safely applied for BDOD management and may provide better outcomes of transplantation by preventing transfusion-related complications. Further randomized controlled trials are needed to investigate an optimal transfusion threshold to improve graft function and to increase the rate of organ procurement.

## Acknowledgements

We thank Sun Kyeong Song, RN, and Eun Kyung Kwon RN for their help with managing data and providing patient care.

## Author contributions

**Conceptualization:** Kyu-Hyouck Kyoung.

**Data curation:** Sungjeep Kim, Kyunghak Choi, Min Soo Kim.

**Formal analysis:** Kyu-Hyouck Kyoung , Min Ae Keum.

**Investigation:** Kyunghak Choi, Sun Geon Yoon.

**Methodology:** Min Ae Keum, Min Soo Kim, Sun Geon Yoon.

**Supervision:** Kyu-Hyouck Kyoung.

**Validation:** Min Ae Keum, Min Soo Kim.

**Writing – original draft:** Sungjeep Kim.

**Writing – original draft & editing:** Sungjeep Kim, Kyu-Hyouck Kyoung.

## Correction

When originally published, figure 1 and 2 were switched and have been corrected.
